# Effect of the COVID-19 pandemic on maternal and neonatal health services in three referral hospitals in Guinea: an interrupted time-series analysis

**DOI:** 10.1186/s12978-023-01599-8

**Published:** 2023-03-25

**Authors:** Tamba Mina Millimouno, Nafissatou Dioubaté, Harissatou Niane, Mamadou Cellou Diallo, Cécé Maomou, Telly Sy, Ibrahima Sory Diallo, Aline Semaan, Thérèse Delvaux, Lenka Beňová, Alexandre Delamou

**Affiliations:** 1 Centre National de Formation et de Recherche en Santé Rurale de Maferinyah, Forécariah, Guinea; 2 Centre d’Excellence d’Afrique pour la Prévention et le Contrôle des Maladies Transmissibles (CEA-PCMT), Faculté des Sciences et Techniques de la Santé, Université Gamal Abdel Nasser de Conakry, Conakry, Guinea; 3 Institut de Nutrition et de Santé de l’Enfant, Hôpital National Donka, Conakry, Guinea; 4 Service de Maternité de l’Hôpital National Ignace Deen, Conakry, Guinea; 5 Service de Maternité de l’Hôpital Régional de Mamou, Mamou, Guinea; 6 Department of Public Health, Institute of Tropical Medicine, Antwerp, Belgium

**Keywords:** Effect, COVID-19, Pandemic, Maternal and neonatal health, Guinea

## Abstract

**Introduction:**

In sub-Saharan Africa, there is limited evidence on the COVID-19 health-related effect from front-line health provision settings. Therefore, this study aimed to analyse the effect of the COVID-19 pandemic on routine maternal and neonatal health services in three referral hospitals.

**Materials and methods:**

We conducted an observational study using aggregate monthly maternal and neonatal health services routine data for two years (March 2019–February 2021) in three referral hospitals including two maternities: Hôpital National Ignace Deen (HNID) in Conakry and Hôpital Regional de Mamou (HRM) in Mamou and one neonatology ward: Institut de Nutrition et de Santé de l’Enfant (INSE) in Conakry. We compared indicators of health service utilisation, provision and health outcomes before and during the COVID-19 pandemic periods. An interrupted time-series analysis (ITSA) was performed to assess the relationship between changes in maternal and neonatal health indicators and COVID-19 through cross-correlation.

**Results:**

During COVID-19, the mean monthly number (MMN) of deliveries decreased significantly in HNID (*p* = 0.039) and slightly increased in HRM. In the two maternities, the change in the MMN of deliveries were significantly associated with COVID-19. The ITSA confirmed the association between the increase in the MMN of deliveries and COVID-19 in HRM (bootstrapped F-value = 1.46, 95%CI [0.036–8.047], *p* < 0.01). We observed an increasing trend in obstetric complications in HNID, while the trend declined in HRM. The MMN of maternal deaths increased significantly (*p* = 0.011) in HNID, while it slightly increased in HRM. In INSE, the MMN of neonatal admissions significantly declined (*p* < 0.001) and this decline was associated with COVID-19. The MMN of neonatal deaths significantly decreased (*p* = 0.009) in INSE and this decrease was related to COVID-19.

**Conclusion:**

The pandemic negatively affected the maternal and neonatal care provision, health service utilisation and health outcomes in two referral hospitals located in Conakry, the COVID-19 most-affected region.

## Introduction

Globally, the COVID-19 pandemic has severely affected almost all sectors [1], compromising the achievement of the Sustainable Development Goal 3 (targets 3.1 and 3.2). In general, infectious disease outbreaks might be harmful to the utilisation and availability of essential reproductive health services [2–6], due to health authorities’ attention being shifted to respond to inevitable shocks, collapse of the health system, or intentional response decisions [7].

Studies report different findings on the effects of the COVID-19 pandemic on maternal and neonatal health services across the world. A study conducted in Nepal in 2020 showed a drastic 50% drop in facility-based deliveries and a significant increase of 27 neonatal deaths per 1000 live births during the first nine weeks of the pandemic [8]. In Zimbabwe, the Ministry of Health and Child Care reported a reduction in the use of maternity services, and the review of maternal care outcomes revealed an increasing trend in maternal deaths during the COVID-19 pandemic [9]. In South Africa, a study carried out in 2020 found a significant 50% drop in hospital admissions for under-five children and a 47% increase in neonatal hospital deaths during the three months following the start of the pandemic [10]. In the same country, another study showed mixed variations in hospital-based deliveries between March and May 2020 during the pandemic [11]. Mixed variation in hospital-based deliveries was also reported in Nigeria during the pandemic [11]. In contrast, other studies have found no adverse effects of COVID-19 on maternal and neonatal health service provision and use [12, 13].

**Fig. 1:**
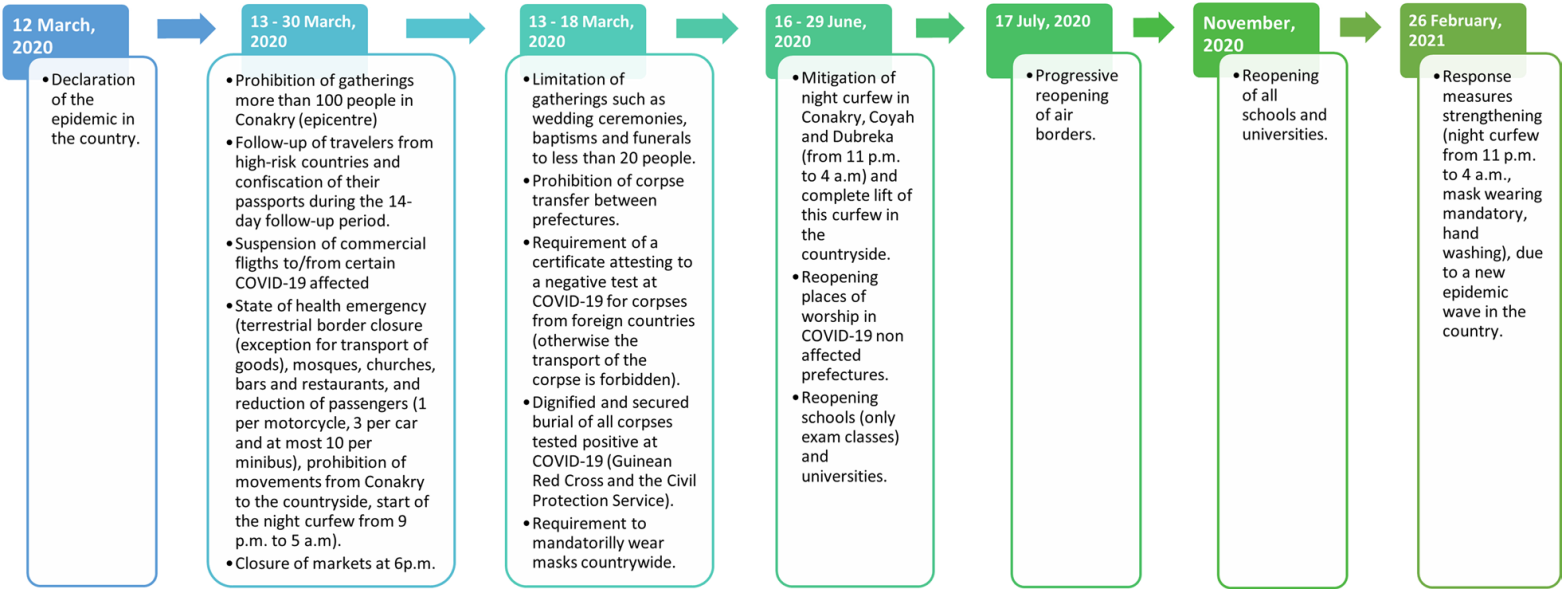
Timeline summarising national authority response measures to restrict the spread of the COVID-19 pandemic in Guinea from 12 March 2020 to 28 February 2021

In Guinea, the COVID-19 outbreak spread upon its start on 12 March 2020 [14]. As of 28 February 2021, 15,894 confirmed cases of COVID-19, of which 89 deaths were reported countrywide [15]. Various response measures, including night-time curfew in the capital city and international travel ban at the national level were put in place (Fig. 1) [16], and new standard operating procedures for the provision of care were established in health facilities, namely in hospitals. These included reducing the number of health personnel by dismissing medical interns, replacing daily face-to-face hospital meetings with online communication groups (e.g. WhatsApp groups), and prioritising providing care to emergency cases over regular routine consultations [17]. Improving maternal and neonatal health is a priority for national health authorities in Guinea [18, 19], which calls the need for identifying how maternal and newborn healthcare provision and health service utilisation were maintained during the COVID-19 pandemic. There is limited knowledge about the effect of the COVID-19 pandemic on maternal and neonatal health services in Guinea [20]. Thus, this study aimed to fill this knowledge gap, in order to inform national health authorities of potential strategies to maintain and improve maternal and neonatal health within a pandemic context.

The main objective of this study was to analyse the effect of COVID-19 on routine maternal and neonatal health services in Guinea. Specifically, in two health regions—Conakry and Mamou, the study sought to: 1) compare indicators of use, provision and outcomes of maternal and neonatal health services one year before and during COVID-19, and 2) analyse trends in service utilisation (deliveries and neonatal admissions), care provision (labour induction) and outcomes (obstetric complications and maternal and neonatal deaths) between the pre-and during-COVID-19 periods.

## Materials and methods

### Study design and period

We conducted an observational study using aggregate monthly routine data of maternal and neonatal health indicators for two years (March 2019–February 2021) in three referral hospitals in Guinea.

### General setting

Guinea, whose capital is Conakry, is a West African country severely affected by the Ebola virus disease between 2014 and 2016. It is one of the poorest countries worldwide and has an estimated population of 12 million inhabitants, of which 52% are women, and 16% are children under-five. Guinea is divided into four natural regions and made up of eight administrative regions and 33 prefectures. The health system is organised into three levels: central (office of the Minister of Health, general secretariat, central directorates, and structures under supervision), intermediate (eight health regions) and peripheral (38 health districts). The health care system is based on health facilities, divided according to their legal status into four sub-sectors (public, mid-public, private, and community) and their package of activities by level and type. The public sub-sector has three levels: central (three national hospitals); intermediate (seven regional hospitals); and peripheral or operational (26 prefectural hospitals, seven communal medical centres including five located in Conakry, 410 health centres and 925 health posts). The mid-public sub-sector has three mining company hospitals, one agricultural company hospital and eight dispensaries. The private sub-sector is composed of profit-making facilities and non-profit-making ones. The community sub-sector consists of community-based services with community health workers and traditional medicine providers.

Maternal and neonatal mortality ratios are estimated at 550 maternal deaths per 100,000 live births and 32 neonatal deaths per 1000 live births (or 48% of infant mortality) [21]. The country has an enormous deficit in human resources for health, with only 98 health workers per 100,000 inhabitants. These human resources are unevenly distributed nationwide, with nearly 52% of health workers residing in Conakry and the surrounding prefectures serving only 15% of the population.

### Specific setting

This study is part of an in-depth mixed-methods multicentric study, conducted in addition to Guinea, in three other sub-Saharan African countries: Uganda, Tanzania and Nigeria [17, 22].

In Guinea, the study took place in two health regions: Conakry and Mamou. In these two health regions, we included three referral hospitals (two maternities: Hôpital National Ignace Deen—HNID, in Conakry and Hôpital Regional de Mamou—HRM, in Mamou and one neonatology ward: Institut de Nutrition et de Santé de l’Enfant—INSE, in Conakry).

The maternity ward of HNID in Conakry is one of the three tertiary referral maternities of the country’s healthcare system, providing care to around 6000 deliveries per year. As for the maternity ward of HRM, it is located about 275 km from Conakry and is an intermediate referral service that performs 3600 deliveries per year on average. INSE is the only national referral service for child health, and it provides 1640 consultations (both impatient and outpatient consultations) per year on average [23]. The COVID-19 pandemic situation between Conakry and Mamou has been hugely different. From March 2020 to February 2021, 14,840 COVID-19 positive cases were recorded nationwide, of which 79% (n = 11,781) occurred in the region of Conakry and 10% (n = 1498) in the region of Mamou [23, 24].

### Study population and sampling

An exhaustive sampling was carried out, including aggregate data on all the routine consultations in the maternal and child health services over the study period.

### Variables

Study variables included monthly aggregate indicators of maternal and neonatal health service utilisation, healthcare provision, and health outcomes.

#### Indicators of health service utilisation

Number of deliveries, number of obstetric referrals received, number of post-abortion care, number of newborns (both outpatients and inpatients) admitted to the neonatology unit (admissions), number of neonatal hospitalisations (inpatients only) in INSE, and number of neonatal referrals received in INSE.

#### Indicators of healthcare provision

Number of labour inductions, number of cesarean sections, number of assisted deliveries (use of the vacuum cup or forceps to speed up and facilitate the expulsion of the foetus), and number of episiotomies.

#### Indicators of health outcomes

Number of obstetric complications (anaemia, uterine rupture, and eclampsia), number of maternal deaths (reported during pregnancy, childbirth and postpartum), number of low-birthweight newborns (< 2500 g), number of stillbirths (fresh and macerated) in maternity wards, and number of neonatal deaths (within the first 28 days of birth) in INSE.

### Data sources and collection procedures

We used a standardised Excel spreadsheet for routine data extraction. Data from maternities were collected from consultation registers, national health information system (SNIS) reports and individual medical records (only in the case of maternal deaths); those concerning newborns were collected from the INSE data centralisation Excel spreadsheet. The data collection lasted nine months (from June 2020 to February 2021). A maternal or neonatal care health professional employed at each hospital collected data monthly at each study site with assistance from the hospital statistician. We ensured data quality control identifying and investigating inconsistencies and extreme values during the data collection period. Additionally, we used the district health information software 2 (DHIS2) to collect data on the number of COVID-19 confirmed cases in the two regions with study sites.

### Data analysis

Data collected using an Excel spreadsheet were imported into R software version 4.0.5 for analysis. Mean monthly numbers (MMN) with standard deviations (SD) were calculated to compare the utilisation of health services, provision of healthcare and related health outcomes before and during COVID-19. We then conducted an interrupted time-series analysis (ITSA) to assess the effect of the COVID-19 pandemic (12 months before, 12 months during) on the use of maternal and neonatal health services, the provision of care and the related care outcomes per month. We used the autocorrelation test to examine the significance of the shifts observed in each time series separately and the cross-correlation coefficient to explore the relationship between COVID-19 (input time series) and maternal and neonatal health service utilisation and provision and care outcomes (output time series). By COVID-19, we mean the monthly number of COVID-19 confirmed cases in the two regions (Conakry and Mamou) sheltering the study sites. As the cross-correlation of time-series requires the time series to be stationary (without the seasonal component) and prewhitened [25, 26], we used the Dickey-Fuller test to check the stationarity (to adjust for seasonality) of our time series by detrending or differencing [25]. Prewhitening allowed us to remove spurious correlations based on temporal dependencies between adjacent values of the input time series and removed these influences from the output time series [25]. The ITSA was performed using the Type II Sum Squares, ANCOVA, Lagged Dependent Variable interrupted times series model [27, 28]. We included a default bootstrap model, which runs 1000 replications of the starting model, with randomly drawn samples to drive the 95% CI bootstrap, adjusted F-value (10% suppression) and *p*-value [28]. This model was fitted by estimating the mean difference of dependent variables (maternal and neonatal health service utilisation, care provision and outcomes) between the interrupted period (COVID-19, March 2020 to February 2021) and the uninterrupted period (March 2019 to February 2020), taking into consideration the lag of the dependent variable and any other specified covariate in the model. The significance level was set at p < 0.05.

### Ethical considerations

This study was approved by the Comité National d’Ethique pour la Recherche en Santé (CNERS) in Guinea (ID: 058/CNERS/20) and the Institutional Review Board at the Institute of Tropical Medicine in Belgium (ID: 1372/20). The data was centralised in a computer secured by a password and accessible only to the research staff.

## Results

### Utilisation of maternal and neonatal health services before COVID-19 (March 2019–February 2020) and during COVID-19 (March 2020–February 2021)

Table 1 shows indicators of utilisation of maternal and neonatal health services before and during COVID-19 at the three study sites. In HNID, the MMN of deliveries significantly decreased from 505 (SD:56) before COVID-19 to 452 (SD:71) during COVID-19 (*p* = 0.039). In HRM, MMN increased insignificantly from 292 (SD:16) in the pre-COVID-19 period to 305 (SD:27) during COVID-19. In the two maternity wards, the MMN of obstetric referrals received increased during the pandemic, and the MMN of post-abortion care decreased. In INSE, the MMN of neonatal admissions significantly declined from 167 (SD:21) before to 124 (SD:25) during the pandemic (*p* < 0.001) (Table 1).

**Table 1:** Comparison of indicators of utilisation of maternal and neonatal health services in three referral hospitals in Guinea between the pre-and during-COVID-19 periods

Indicators of utilisation of maternal and neonatal health services	Monthly mean (SD) Pre-COVID-19 (03/2019 – 02/2020)	Monthly mean (SD) During COVID-19 (03/2020 – 02/2021)	*p*-value
*Maternal care*			
HNID			
Deliveries (vaginal and cesarean section)	505 (56)	452 (71)	0.039
Obstetric referrals received	142 (19)	147 (18)	0.513
Post-abortion care	9 (4)	8 (5)	0.437
HRM			
Deliveries (vaginal and cesarean section)	292 (16)	305 (27)	0.400
Obstetric referrals received	13 (4)	16 (8)	0.265
Post-abortion care	9 (3)	7 (3)	0.291
*Neonatal care*			
INSE			
Neonatal admissions	167 (21)	126 (25)	< 0.001

*HNID*  Hôpital National Ignace Deen, *HRM*  Hôpital Régional de Mamou, *INSE*  Institut de Nutrition et de Santé de l’Enfant, *SD*  standard deviation

### Provision of maternal and neonatal healthcare before COVID-19 (March 2019–February 2020) and during COVID-19 (March 2020–February 2021)

Table 2 depicts the comparison of the provision of maternal and neonatal healthcare before and during COVID-19 at the three study sites. The MMN of labour induction increased significantly in the two maternity wards, from 9 (SD:4) before to 16 (SD:4) during COVID-19, in HNID (*p* < 0.001), and from 6 (SD:3) before to 12 (SD:7) during the pandemic, in HRM (*p* = 0.005). The MMN of cesarean sections significantly decreased over the pandemic period in HNID (*p* = 0.04), while it slightly increased in HRM. The MMN of assisted deliveries (using vacuum cup or forceps) significantly decreased over the pandemic period in HNID (*p* = 0.031), while it increased in HRM.

**Table 2:** Comparison of the provision of maternal and neonatal healthcare in three referral hospitals in Guinea between the pre-and during-COVID-19 periods

Indicators of provision of maternal and neonatal healthcare	Monthly mean (SD) Pre-COVID-19 (03/2019 – 02/2020)	Monthly mean (SD) During COVID-19 (03/2020 – 02/2021)	*p*-value
*Maternal care*			
HNID			
Labour induction	9 (4)	16 (4)	0.002
Cesarean sections	241 (8)	228 (12)	0.040
Assisted deliveries by vacuum cup/forceps	11 (5)	7 (5)	0.031
Episiotomies	58 (20)	49 (15)	0.072
HRM			
Labour induction	6 (3)	12 (7)	0.009
Cesarean sections	86 (8)	89 (15)	0.700
Assisted deliveries by vacuum cup/forceps	0 (0)	2 (3)	0.064
Episiotomies	15 (4)	16 (5)	0.563
*Neonatal care*			
INSE			
Neonatal hospitalisations	123 (23)	87 (24)	0.001

*HNID*  Hôpital National Ignace Deen, *HRM*  Hôpital Régional de Mamou, *INSE*  Institut de Nutrition et de Santé de l’Enfant, *SD*  standard deviation

The MMN of neonatal hospitalisations in INSE dropped substantially, from 123 before COVID-19 to 87 during the pandemic (*p* = 0.001) (Table 2).

### Maternal and neonatal health outcomes before and during COVID-19

Table 3 presents the comparison of maternal and neonatal care outcomes before and during COVID-19. The MMN of obstetric complications slightly increased from 21 (SD:6) before to 24 (SD:6) during the pandemic in HNID, while it was similar over the pre and during-COVID-19 periods in HRM. The MMN of maternal deaths increased significantly from 2 (SD:0.6) before to 5 (SD:0.8) during the pandemic (*p* = 0.011) in HNID, while it slightly increased from 1 (0.3) before to 1.5 (0.3) during the pandemic in HRM. The MMN of neonatal deaths significantly decreased from 59 before COVID-19 to 35 over the pandemic period (*p* = 0.009) in INSE (Table 3).

**Table 3:** Comparison of maternal health outcomes (obstetric complications, maternal deaths, low-birthweight, stillbirth) and neonatal care outcome (deaths) in three referral hospitals in Guinea between the pre-and during-COVID-19 periods

Indicators of maternal and neonatal care outcomes	Monthly mean (SD) Pre-COVID-19 (03/2019 – 02/2020)	Monthly mean (SD) During COVID-19 (03/2020 – 02/2021)	*p*-value
*Maternal care outcomes at Ignace Deen maternity*			
Obstetric complications	21 (6)	24 (6)	0.269
Maternal deaths during pregnancy, delivery, and PP	2 (0.6)	5 (0.8)	0.011
Low-birthweight newborns	82 (7)	88 (8)	0.570
Fresh stillbirths	24 (2)	24 (1)	1.000
Macerated stillbirths	24 (2)	23 (2)	0.673
*Maternal care outcomes at Mamou maternity*			
Obstetric complications	40 (3)	40 (2)	0.846
Maternal deaths during pregnancy, delivery, and PP	1 (0.3)	1.5 (0.3)	0.297
Low-birthweight newborns	15 (2)	22 (3)	0.075
Fresh stillbirths	10 (1)	7 (1)	0.156
Macerated stillbirths	16 (1)	17 (1)	0.302
*Neonatal care outcome at INSE*			
Neonatal deaths at INSE	59 (20)	35 (15)	0.009

### Trends in service utilisation (deliveries and neonatal admissions), care provision (labour induction) and outcomes (obstetric complications and maternal and neonatal deaths) between the pre-and during-COVID-19 periods

**Fig. 2:**
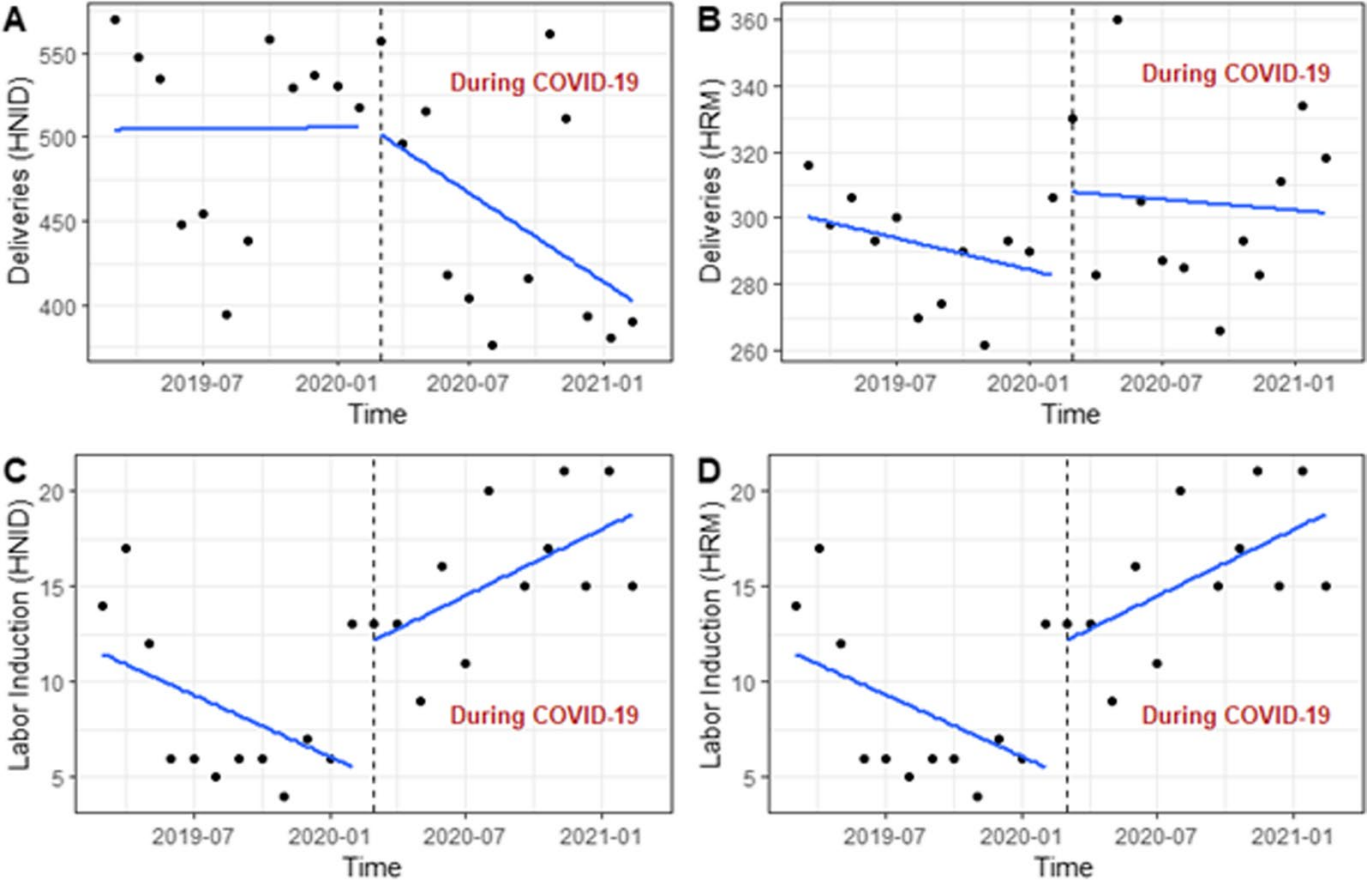
Trends in maternal health service utilisation (deliveries) and provision (labour induction) in two referral hospitals in Guinea between the pre-and during-COVID-19 periods

**Fig. 3:**
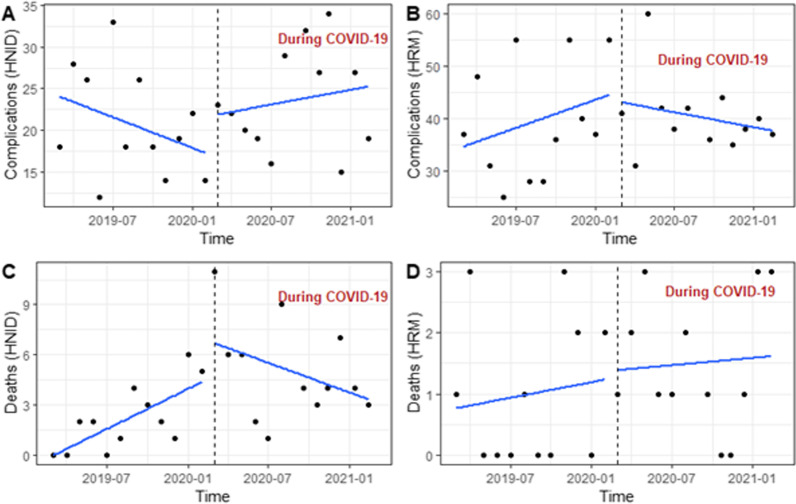
Trends in maternal care outcomes (obstetric complications and maternal deaths) in two referral hospitals in Guinea between the pre- and during-COVID-19 periods

**Fig. 4:**
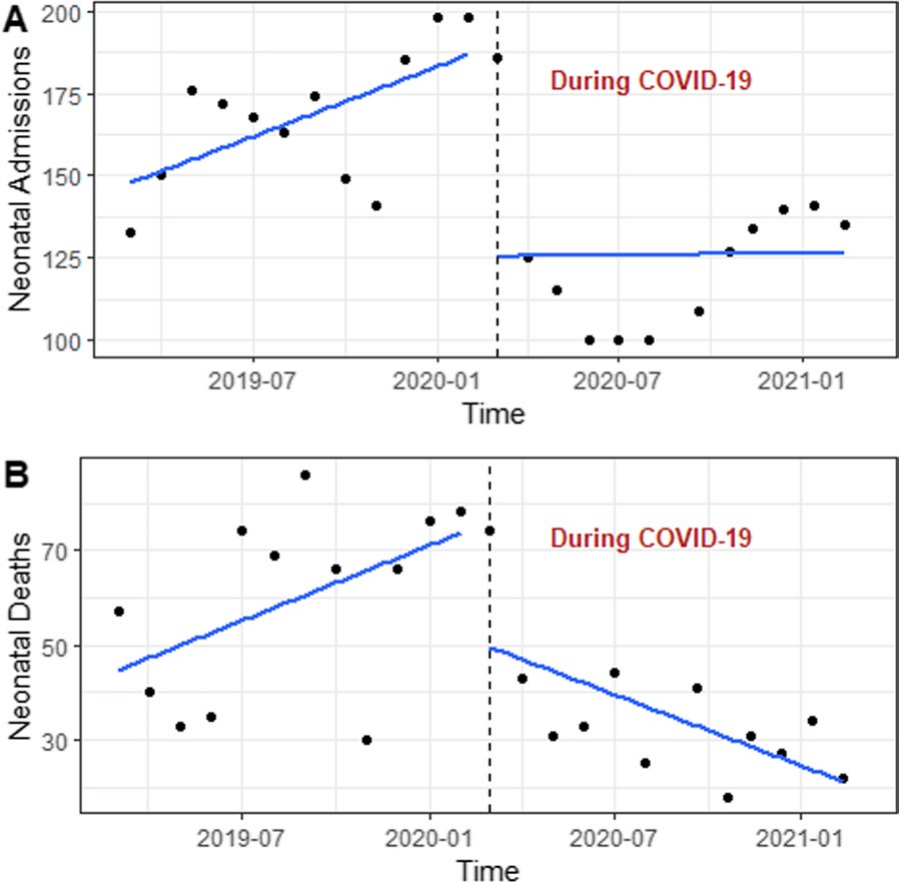
Trends in neonatal health service utilisation (admissions) and care outcomes (deaths) at the Institut de Nutrition et de Santé de l’Enfant (INSE) in Guinea between the pre-and during-COVID-19 periods

In HNID, we observed a slight upward trend in deliveries in the pre-COVID-19 period, while the trend was substantially downward during the pandemic (Fig. 2A). In HRM, deliveries showed a downward trend in the pre-pandemic period and significantly increased after the onset of the pandemic while keeping a slight downward trend over the pandemic period (Fig. 2B). Labour induction has similar shifts in the two maternities before and during the pandemic; the trends were downward in the pre-COVID-19 periods, but immediately after the start of the pandemic, the MMN of labour induction significantly increased while keeping the same direction during the COVID-19 period (Fig. 2C, D). Over the pandemic, we observed an increasing trend in obstetric complications in HNID (Fig. 3A), while this trend declined in HRM (Fig. 3B). Maternal deaths that increased upon the onset of the pandemic present a decreasing trend over the pandemic in HNID (Fig. 3C). In HRM, maternal deaths showed an upward trend over the pandemic (Fig. 3D). In INSE, the number of neonatal admissions had a significant upward trend in the pre-COVID-19 period, but they declined significantly upon the start of the pandemic and stayed relatively stable throughout the pandemic (Fig. 4A). Neonatal deaths dropped drastically immediately after the start of the pandemic, with a downward trend observed over the pandemic period (Fig. 4B).

### Cross-correlation between COVID-19 and indicators of service utilisation (deliveries and neonatal admissions), care provision (labour induction) and outcomes (obstetric complications and maternal and neonatal deaths) during the pandemic

**Fig. 5:**
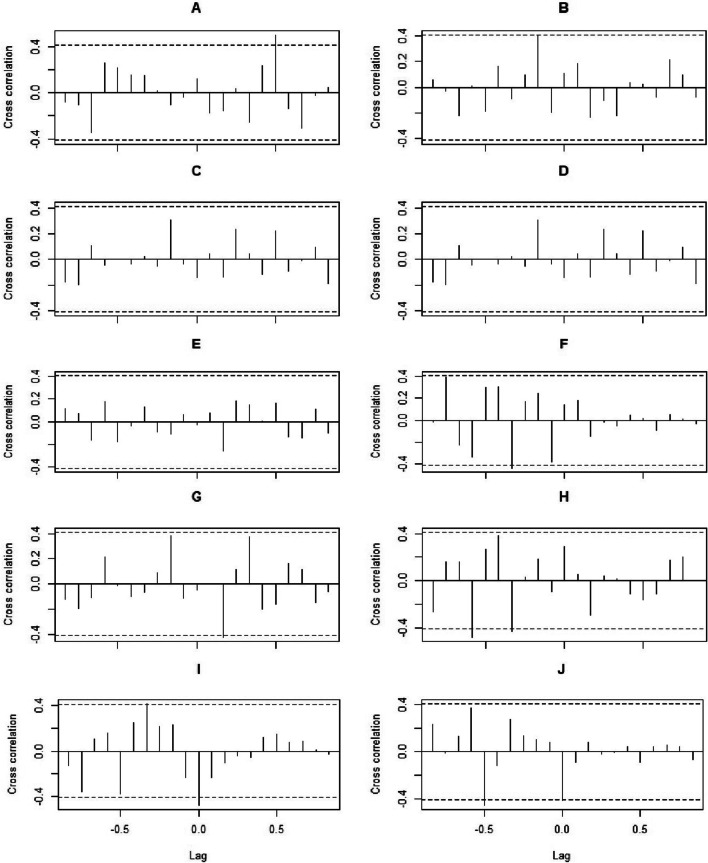
Cross correlations between COVID-19 (number of confirmed cases) time series and the number of deliveries in Hôpital National Ignace Deen (HNID) (**A**) and Hôpital Régional de Mamou (HRM) (**B**), the number of labour induction in HNID (**C**) and HRM (**D**), the number of obstetric complications in HNID (**E**) and HRM (**F**), the number of maternal deaths in HNID (**G**) and HRM (**H**), the number of neonatal admissions (**I**) and the number of neonatal deaths (**J**) in the Institut de Nutrition et de Santé de l’Enfant

The cross-correlation showed significant associations between deliveries and COVID-19 in both HNID (Fig. 5A) and HRM (Fig. 5B). It revealed upward sloping associations between labour induction and COVID-19 in the two maternities (Figs. 5C, D). There was no correlation between obstetric complications and COVID-19 in HNID (Fig. 5E), while they correlated in HRM (Fig. 5F). Maternal deaths and COVID-19 significantly correlated in both HNID (Fig. 5G) and HRM (Fig. 5H). The decrease in the number of neonatal admissions in INSE during the COVID-19 pandemic was significantly associated with COVID-19 confirmed cases (Fig. 5I). The cross-correlation test revealed a significant relationship between COVID-19 confirmed cases and neonatal deaths (Fig. 5J).

### The ITSA of indicators of service utilisation, care provision and outcomes between the pre-and during-COVID-19 periods

The ITSA supported the associations between—the increase in the number of deliveries in HRM and the low number of COVID-19 confirmed cases in the region of Mamou (bootstrapped F-value = 1.5, 95%CI [0.036–8.047], *p* = 0.01)—the increase in the number of labour induction in HNID and the increase in the number of COVID-19 confirmed cases in the region of Conakry (bootstrapped F-value = 8.9, 95%CI [0.206–18.290], *p* < 0.01)—the slight increase in maternal deaths in HRM and the low number of COVID-19 confirmed cases in Mamou (bootstrapped F-value = 1.4, 95%CI [0.14 – 10.49], *p* = 0.035) (Table 4).

**Table 4:** Results of the interrupted time series analysis model of maternal and neonatal healthcare services during COVID-19 in three referral hospitals in Guinea

Parameters	Sum Sq	Df	F-value	*p*-value
*Number of deliveries at Hôpital National Ignace Deen (HNID)*				
COVID-19 period	3483	1	1.131	0.300
Lag_deliveries	23,242	1	7.551	0.012*
Bootstrapped F-values (95% CI)			1.384 (0.176–9.806)	0.272
*Number of deliveries at Hôpital Régional de Mamou (HRM)*				
COVID-19 period	17	1	1.012	0.326
Lag_deliveries	251	1	14.599	< 0.01**
Bootstrapped F-values (95% CI)			1.462 (0.036–8.047)	< 0.01**
*Number of labour induction at HNID*				
COVID-19 period	84.813	1	5.763	0.026*
Lag_labour induction	32.703	1	2.222	0.151
Bootstrapped F-values (95% CI)			8.920 (0.206–27.642)	0.032*
*Number of labour induction at HRM*				
COVID-19 period	1027	1	2.037	0.168
Lag_labour induction	96	1	0.191	0.667
Bootstrapped F-values (95% CI)			1.561 (0.159–18.290)	0.060
*Number of obstetric complications at HNID*				
COVID-19 period	52	1	1.264	0.274
Lag_obstetric complications	36	1	0.883	0.358
Bootstrapped F-values (95% CI)			1.3086 (0.060–8.428)	0.170
*Number of obstetric complications at HRM*				
COVID-19 period	1027	1	2.037	0.168
Lag_obstetric complications	96	1	0.190	0.667
Bootstrapped F-values (95% CI)			1.561 (0.159–18.290)	0.061
*Number of maternal deaths at HNID*				
COVID-19 period	20.624	1	3.141	0.091
Lag_maternal deaths	1.227	1	0.186	0.670
Bootstrapped F-values (95% CI)			2.006 (0.005–7.418)	0.191
*Number of maternal deaths at HRM*				
COVID-19 period	1.009	1	0.707	0.410
Lag_ maternal deaths	0.474	1	0.332	0.570
Bootstrapped F-values (95% CI)			1.366 (0.141–10.491)	0.035*
*Number of neonatal admissions at Institut de Nutrition et de Santé de l'Enfant (INSE)*				
COVID-19 period	2595	1	10.024	0.01**
Lag_neonatal admissions	5010	1	19.350	< 0.001***
Bootstrapped F-values (95% CI)			8.047 (0.502–48.267)	0.577
*Number of neonatal deaths at INSE*				
COVID-19 period	1290	1	4.934	0.038*
Lag_ neonatal deaths	1384	1	5.292	0.032*
Bootstrapped F-values (95% CI)			5.973 (0.019–25.661)	0.209

*HNID*  Hôpital National Ignace Deen, *HRM*  Hôpital Regional de Mamou, *INSE*  Institut de Nutrition et de Santé de l’Enfant, *CI* Confidence Interval, *Sum Sq*  sum of squares, *Df*  degrees of freedom, *F*  Fisher's test; *p < 0.05; **p < 0.01; ***p < 0.001

## Discussion

To our knowledge, this study is the first of its kind to quantitatively analyse the effect of the COVID-19 pandemic on maternal and newborn health services in Guinea.

Overall, the study showed a negative trend in the provision of maternal and neonatal healthcare and utilisation of maternal and neonatal health services and the resulting health outcomes in Conakry, the pandemic epicentre in Guinea. In contrary, it did not find any negative variation in the studied maternal and neonatal indicators in Mamou, the COVID-19 least-affected region.

Deliveries had a mixed trend during COVID-19 in the two maternity wards included in the study. They decreased in HNID in Conakry, while they increased upon the onset of the pandemic, then keeping a slightly downward trend in HRM located in a less COVID-19 affected area. Indeed, our findings show that the decrease in the number of deliveries in HNID was associated with the level of spread of COVID-19 in Conakry, against which response measures were put in place, including transport restrictions. These transport restrictions, the women’s increased anxiety about or fear of contracting COVID-19 in health facilities or the possibility of transmission of the disease to their babies, and resource constraints in the COVID-19 context would have discouraged some pregnant women, especially those with low-risk pregnancies (without complications), from giving birth in HNID, opting for smaller local health facilities (communal medical centres, health centres and private health facilities) or to give birth at home [22]. Several authors have already mentioned at least one of these factors to have contributed to the reduction in the use of health services during the COVID-19 pandemic [13, 29–33]. It is vital that pregnant women give birth in an appropriate setting with skilled birth attendants and life saving equipment to improve maternal and neonatal health, by treating complications during delivery, and reduce preventable maternal and newborn morbidity and mortality, and stillbirths. However, in Mamou, where the pandemic started late with relatively slow growth, we have seen an increase in the number of deliveries in the maternity of the regional hospital. This result could be explained by the fact that this region was much less affected by COVID-19 than Conakry, and therefore, there was few anxieties related to the pandemic in the community, ample freedom for movement due to somewhat less restrictive response measures, which could have promoted the use of health services. The transportation ban from Conakry might have also played a role in this positive effect because it would have prevented some pregnant women, especially those with complex or high-risk pregnancies, from giving birth in Conakry, for fear of not being able to come back home (Mamou) early after childbirth. Similar findings were drawn by Ahmed et al. and Abdela et al., who respectively reported a negative trend in deliveries during the pandemic at Aminu Kano university teaching hospital located within a COVID-19 heavily affected State in Nigeria [11], and a relatively stable trend in deliveries over the pandemic period at Dessie referral hospital situated in a catchment area slightly affected by the pandemic in Ethiopia [34].

The number of labour inductions performed significantly increased during the COVID-19 period in the two maternity wards. This was probably an intentional practice to—speed up labour and reduce the amount of time women spend in the facility—lower women fear of nosocomial infection—avoid crowding and maintain social distancing in a context of lack of space in crowded maternities. However, inducing labour is not recommended when it is not essential, as it carries many unnecessary risks such as low fetal heart rate, infection, uterine rupture and bleeding after delivery.

Obstetric complications revealed a mixed pattern. They increased upon the start of the COVID-19 pandemic while maintaining an upward trend during the pandemic in HNID. This trend might be attributable to the increase in obstetric referrals received from primary healthcare facilities in Conakry. This could additionally be explained by potential delays in the decision to seek care and in reaching the healthcare facilities due the COVID-19 related restriction measures and community perceptions; leading women to arrive late to the facilities after the severity of obstetric complications had amplified. Although obstetric complications were increasing during the pre-COVID-19 period, they showed a downward trend during the pandemic in Mamou—HRM. This downward trend could probably be due, on the one hand, to the improvement in the quality of obstetric care through the new care-guidelines established in the referral health services within the context of COVID-19, especially the prioritisation of essential care to routine ones, and an increased focus on infection prevention and control, and on the other hand, to the rapid decision in reaching healthcare facilities, as women were emotionally less affected by the pandemic, with the very few confirmed cases in the Mamou region.

Maternal mortality also depicted a mixed pattern. it rose sharply in HNID as soon as COVID-19 was declared in the country, with maternal deaths trending down. This increase in maternal mortality upon the start of the pandemic and then controlled by healthcare providers over the pandemic might result from the increase in the number of obstetric complications in this maternity, as shown by our results. In addition, it could result from the delay in decision to seek and reaching care associated with various aspects of COVID-19, and it might also be due to the lack of ability to seek antenatal care (ANC). In addition, our findings show that the decline in maternal deaths over the COVID-19 period in HRM followed the slow growth in the pandemic whose control measures were minimal and may not have prevented pregnant women from regularly attending the ANC visits or seeking care in a timely manner. This could possibly explain that maintaining ANC schedules in Mamou resulted in the downward trend in obstetric complications, as shown by our findings.

Neonatal admissions to the neonatology ward of INSE in Conakry experienced a significant sharp decrease from the start of the pandemic, pursuing throughout the pandemic. Chimhuya et al. found corroborating results in Zimbabwe and Malawi [35]. They reported that neonatal admissions to the neonatology unit at Kamuzu Central Hospital in Malawi declined significantly upon the first week of the pandemic. In contrast, they found no significant decline in neonatal admissions from the start of the pandemic at the neonatology unit at Sally Mugabe Central Hospital in Zimbabwe. In our context, the decrease in use of the neonatal referral service could also be connected with the factors already mentioned above, particularly transport restrictions, the women’s increased anxiety about or fear of contracting COVID-19 in health facilities or the possibility of transmission of the disease to their babies, and resource constraints.

Our study also found that neonatal deaths fell significantly in INSE upon the announcement of the pandemic while maintaining a downward trend during the pandemic. This trend is proportional to the significant reduction in neonatal admissions to the INSE during the pandemic, on the one hand, and to the capacity strengthening of health staff in managing respiratory diseases, particularly for newborns within the COVID-19 context, on the other hand. Nirajana et al. reported a different result in Nepal; neonatal deaths increased with increasing neonatal admissions during the pandemic [36]. Our finding are also opposite to that reported by Chimhuya et al. in Zimbabwe and Malawi where a slight weekly increase in neonatal deaths over the pandemic period was observed [35].

### Study strengths and limitations

This study has the merit of having been conducted within two hugely different pandemic contexts in Guinea, Conakry which was heavily affected and Mamou, whereby the number of cases was minimal. This allowed us to adopt a comparative analytical lens taking structural elements related to the pandemic into consideration when interpreting our findings. In addition, it is noteworthy that, as the current study is part of a mixed methods study, it is complemented by a qualitative strand which explored healthcare providers’ perceptions and experiences of the COVID-19 response in the three referral hospitals, and the paper that reports on that qualitative strand has been submitted elsewhere for publication [37]. However, this study includes some limitations. First, it was conducted in only three referral hospitals; and is therefore only representative of these three healthcare facilities and the findings are not generalisable to the larger number of health facilities in Guinea. Second, we did not collect information on the reasons behind the decrease in patient flow and whether patients attended other healthcare facilities or delivered at home. Third, we did not collect data from women about their perceptions and healthcare seeking behaviours before and during the pandemic. In addition, we relied on secondary routine data which might have some quality issues (e.g., missingness). Therefore, a further wider multi hospital-based and community-based study is needed to better understand the dynamics of maternal and newborn healthcare service provision, use, and outcomes in the context of the COVID-19 pandemic.

## Conclusion

Our study showed that the COVID-19 pandemic negatively affected the use and provision of maternal and neonatal health services and the resulting outcomes on the two study sites located in Conakry, within the region most affected by the pandemic. There is a need for national health authorities and local partners to focus on accessibility to quality maternal and neonatal health services within a pandemic context by designing parallel strategies and interventions to counter service disruptions and reduce preventable maternal and perinatal deaths. Particularly, preparedness to health system shocks by developing toolkits to use for monitoring health service disruptions and adjusting programs accordingly should be given a priority.

## Data Availability

All data generated or analysed during this study are included within this published article.

## Abbreviations

ANCOVA: Analysis of covariance
ANC: Antenatal care
MMN: Mean monthly number
CNERS: Comité National d’Ethique pour la Recherche en Santé
COVID-19: Coronavirus disease of 2019
DHIS2: District Health Information Software 2
INSE: Institut de Nutrition et de Santé de l’Enfant
SD: Standard deviation
HNID: Hôpital National Ignace Deen
HRM: Hôpital Regional de Mamou
